# Expanding the Material Search Space for Multivalent Cathodes

**DOI:** 10.1021/acsami.2c11733

**Published:** 2022-09-22

**Authors:** Ann Rutt, Jimmy-Xuan Shen, Matthew Horton, Jiyoon Kim, Jerry Lin, Kristin A. Persson

**Affiliations:** † Department of Materials Science and Engineering, University of California, Berkeley California 94720, United States; ‡ Materials Sciences Division, Lawrence Berkeley National Laboratory, Berkeley California 94720, United States

**Keywords:** computational screening, cathodes, multivalent batteries, energy storage, density functional theory, high-throughput

## Abstract

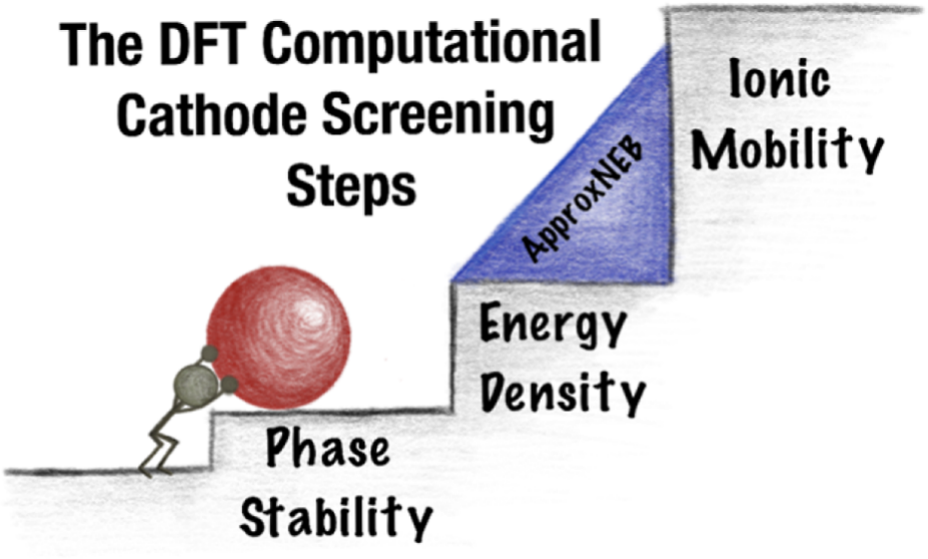

Multivalent batteries are an energy storage technology with the potential to surpass lithium-ion batteries; however, their performance have been limited by the low voltages and poor solid-state ionic mobility of available cathodes. A computational screening approach to identify high-performance multivalent intercalation cathodes among materials that do not contain the working ion of interest has been developed, which greatly expands the search space that can be considered for material discovery. This approach has been applied to magnesium cathodes as a proof of concept, and four resulting candidate materials [NASICON V_2_(PO_4_)_3_, birnessite NaMn_4_O_8_, tavorite MnPO_4_F, and spinel MnO_2_] are discussed in further detail. In examining the ion migration environment and associated Mg^2+^ migration energy in these materials, local energy maxima are found to correspond with pathway positions where Mg^2+^ passes through a plane of anion atoms. While previous studies have established the influence of local coordination on multivalent ion mobility, these results suggest that considering both the type of the local bonding environment and available free volume for the mobile ion along its migration pathway can be significant for improving solid-state mobility.

## Introduction

Multivalent (e.g., Mg, Ca, and Zn) batteries have been explored as a “beyond Li-ion” technology but are currently limited in their promise as high-energy-density batteries due to the lack of suitable electrolytes and cathodes.^1−3^ Among the most promising recent advancements in Mg cathodes are spinel Mg_*x*_Ti_2_S_4_^4,5^ and layered Mg_*x*_TiS_2_,^4,6^ which show improved capacities (160 mA h/g for spinel Mg_*x*_Ti_2_S_4_ and 140 mA h/g for layered Mg_*x*_TiS_2_) compared to the original Mg cathode, Chevrel Mg_*x*_Mo_6_S_8_ (100 mA h/g).^7^ However, the spinel Mg_*x*_Ti_2_S_4_ and the layered Mg_*x*_TiS_2_ cathodes share two limitations that must be overcome in order to realize high-performance magnesium batteries: (1) low voltages (1.2 V vs Mg/Mg^2+^) and (2) poor solid-state mobility (requiring elevated cycling temperatures of 60 °C).

Calcium battery research is still in its early stages, especially in comparison to Mg-based systems, but there have been recent advances in reversible Ca plating.^8−10^ Some promising Ca cathode candidates such as Na_0.5_VPO_4.8_F_0.7_ (NVPF)^11^ and the sodium superionic conductor (NASICON) NaV_2_(PO_4_)_3_^12,13^ have shown capacities of 87 and 83 mA h/g, respectively. Compared to their Mg counterparts, these compounds show higher voltages of ∼3.2 V versus Ca/Ca^2+^ and slighter lower Ca^2+^ intercalation capacities. More recently, CaV_2_O_4_ was identified as a potential Ca cathode with computational methods and shown to cycle up to 10 cycles at 50 °C, albeit with notable cell polarization.^14^ Despite these advances, it has proved difficult to identify a high voltage and high capacity Ca cathode that performs well under ambient conditions with repeated cycling.

Computational methods that use density functional theory (DFT) to evaluate cathode properties of interest such as phase stability, voltage, capacity, and ionic diffusivity have been well established.^15−17^ In the past 10–15 years, researchers began combining these methods in high-throughput computational screenings to evaluate various materials as cathodes. For example, in 2011, 277 mixed polyanionic compounds of the sidorenkite structure type were computationally screened as lithium and sodium cathodes where phase stability, voltage, and capacity properties were reported.^18^ Follow-up work focused on carbonophosphates Li_3_MPO_4_CO_3_ (M = transition metal) as a novel family of Li-ion cathode materials, which also included the investigation of lithium mobility in one compound, Li_*x*_Mn(PO_4_)(CO_3_), at various lithium concentrations (*x* = 1, 2, 3).^19^ There has also been interest in computationally screening sulfide and oxide spinels as multivalent cathodes where properties such as phase stability, voltage, and capacity were evaluated for ∼60 compositions.^20,21^ These properties were used to down select a smaller subset of 7 spinel compositions for investigating the cation mobility. The computational exploration of six perovskites as Ca cathodes resulted in evaluating the Ca mobility in one composition identified as the most promising perovskite compound.^22^ A high-throughput computational screening methodology for layered materials has also been reported for sodium and multivalent cathodes, which included the calculation of migration barriers for ∼40 down selected layered materials.^23,24^ A recent screening of polyanionic materials as K-ion cathodes used computational methods to down select candidate materials based on composition, stability, capacity, and voltage, at which point, four compounds were selected for experimental investigation.^25^ Potassium mobility was computationally investigated for one compound.

Across these efforts, there has been a fundamental limitation insomuch that only one structural family, where the diffusion pathway and intercalation sites are already known, is considered. Recent work includes consideration of a wider range of structure types for Li-ion cathodes^26^ and Mg-ion cathodes;^27^ however, these materials all contained the working ion of interest where the intercalation sites are known.

Cathode computational screenings that consider ionic mobility have typically used nudged elastic band (NEB) calculations in conjunction with DFT to estimate the migration barrier.^20,21,23,24,26,28^ NEB calculations are notoriously challenging due to the domain expertise required to inform the calculation inputs, their high computational cost, and numerical sensitivity that requires careful inspection of the calculation outputs. These challenges are evidenced by the number of cathode computational screenings that reserve performing NEB calculations for the single, most promising candidate,^19,22,26^ exclude ionic mobility entirely,^17,18^ or opt to pursue experimental electrochemical testing before performing a NEB calculation.^25^

Given the challenges with NEB calculations, there have been several studies dedicated to acceleration efforts. The most common strategy to lower computational cost is reducing the number of image relaxations along the predicted pathway. For example, some algorithms start with a lower image resolution that is iteratively increased such as in ANEBA^29^ and AutoNEB.^30^ In R-NEB, redundant image relaxations are avoided by using the system’s reflection symmetry.^31^ Machine learning has also been applied to approximate potential energy surfaces to more efficiently find the minimum energy pathway.^32−34^ Compared to these approaches, which rely on iterative interconnected image relaxations, ApproxNEB^35^ is unique in that it evaluates images as single-point calculations after an improved path initialization based on the material charge density.

The challenge of identifying multivalent intercalation cathodes with good solid-state mobility correlates directly to their potential for higher capacity.^1^ While the higher valence of multivalent ions can lead to a higher energy density, there is also a trade-off associated with poor mobility due to the stronger Coulombic interactions between the mobile multivalent ion and the surrounding cathode host lattice. Previous work^36^ has shown that multivalent ion mobility correlates with the local coordination topology along the diffusion path, favoring flat electrostatic landscapes with no strong binding sites for the multivalent ion. For example, materials which contain Mg in their as-synthesized states tend to exhibit relatively strong Mg-binding sites and hence poorer mobility.

Interestingly, the most successful Mg cathodes have originated from compounds synthesized in a form without Mg. In the case of Chevrel Mg_*x*_Mo_6_S_8_, the copper-containing CuMo_3_S_4_ is synthesized, and the copper then chemically removed.^37^ Similarly, for spinel Mg_*x*_Ti_2_S_4_, CuTi_2_S_4_ is synthesized, and then, the copper removed by oxidation.^5^ Layered Mg_*x*_TiS_2_ can be synthesized to be free of any intermediate compound as TiS_2_.^6^ The NVPF and NASICON Ca cathodes discussed previously were also originally synthesized without Ca. Na_1.5_VPO_4.8_F_0.7_ and NASICON Na_3_V_2_(PO_4_)_3_ are synthesized and then partially electrochemically desodiated to obtain Na_0.5_VPO_4.8_F_0.7_ and NaV_2_(PO_4_)_3_,^11−13^ which can cycle as Ca cathodes. Recently, CaV_2_O_4_ became the first Ca cathode to cycle 10 times that was synthesized in the discharged state by performing a solid-state reaction to obtain CaV_2_O_6_, which is subsequently reduced to CaV_2_O_4_.^14^ A successful Mg cathode which has been synthesized in the discharged state (where the Mg ion is contained in the structure) is yet to be reported.^1^

Hence, one may extrapolate that identifying new multivalent cathodes among materials that already contain the multivalent ion of interest would yield scarce results. An automated computational infrastructure for discovering intercalation electrodes has been previously reported;^27^ however, these efforts have focused exclusively on evaluating materials in the discharged state where the intercalation sites are already known. Given the solid-state mobility challenges with multivalent ions, new strategies are needed to discover high-performance multivalent cathodes. In this paper, we report a new comprehensive computational framework for identifying novel multivalent cathode materials in compounds that do not a priori contain the working ion, for example, they are most stable in their charged state. This framework uses for the first time a combined methodology of the insertion algorithm^38^ and the ApproxNEB algorithm^6^ to screen by solid-state mobility properties in an automated high-throughput manner. We demonstrate our approach to computationally screen thousands of materials from the Materials Project database^39^ in order to evaluate promising Mg cathodes. From this screening, four materials are highlighted and discussed in further detail. The automatic computational evaluation of two of these materials is consistent with previously reported experiments testing their electrochemical properties as Mg cathodes which validates this screening framework. Furthermore, NASICON V_2_(PO_4_)_3_ and birnessite NaMn_4_O_8_ are identified as potential promising Mg cathodes for further consideration.

## Methods

**Figure 1:**
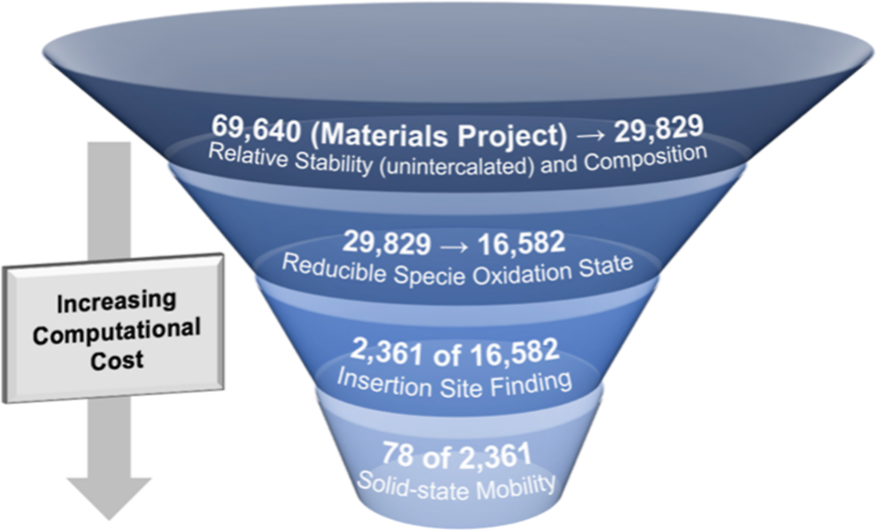
Funnel graphic summarizing the screening process for finding promising multivalent cathodes among materials in the deintercalated state. The screening process has been divided into four stages in order of increasing computational cost. The number of materials considered in each tier has been included for the described work on Mg cathodes to clarify the screening process and its limitations. However, we note that the exact numbers will change if the methodology is applied today as the number of materials in the Materials Project has almost doubled.

The presented computational approach for evaluating materials free of the working ion of interest as multivalent cathodes can be described in four screening tiers. The criteria for each tier were ordered by a combination of robustness and computational cost. Less computationally demanding methods, which still correlate well to experimental results, were used first in earlier tiers, in order to limit applying more expensive methods to a smaller number of candidate materials. Each tier focused on a different set of material properties, which are depicted in Figure 1, which will be described in more detail:

1. Relative stability and composition
2. Reducible specie oxidation state
3. Insertion site finding
4. Multivalent ion solid-state mobility

### Relative Stability and Composition

The Materials Project, a database of DFT calculations of inorganic crystals and molecules, was used to source candidate materials.^39^ We note that the examination of Mg-free (“empty”) hosts presents a vast space of many tens of thousands of possible materials. Two properties, relative stability and composition, were used to narrow down this search space. Relative stability compared to other materials composed of the same elements was captured by the quantity, energy per atom above the convex hull, which encloses the most stable phases within the relevant chemical space.^40,41^ This “energy above hull” is 0 eV/atom for a material that is predicted to be thermodynamically stable at 0 K. A cut-off value of <0.2 eV/atom was used to select compounds that are likely to be synthesizable based on previous work that established sensible cut-off values.^42,43^ Composition constraints were also applied that required the candidate material: (1) to contain at least one redox active element (Ti, V, Cr, Mn, Fe, Co, Ni, Cu, Nb, Mo, Ru, Ag, W, Re, Sb, and Bi), (2) to contain either oxygen or sulfur, and (3) excludes radioactive elements (elements with an atomic number between 84 and 104). Using these criteria, the Materials Project database (version 2018.11), which contained 69,640 inorganic crystals at the time that this work was started, was reduced to 29,829 candidate materials of interest.

### Reducible Specie Oxidation State

Given the intention to insert magnesium into empty candidate host materials, viable candidates need to encompass a reducible specie that can accept electrons upon Mg insertion. The next screening tier is focused on finding materials with a high enough oxidation state to permit reduction. There are two algorithms within pymatgen^44^ that are able to suggest likely oxidation states of a given material. One uses a bond valence method^45^ to determine oxidation states and the other predicts appropriate oxidation states based on the material’s chemical formula using a data mining approach. Candidate materials were discarded at this tier if they did not contain any of the following reducible species: Ti^4+^, V^4+,5+^, Cr^4+,5+,6+^, Mn^3+,4+,5+,6+,7+^, Fe^3+,4+,5+,6+^, Co^3+,4+,5+^, Ni^3+,4+^, Cu^2+,3+,4+^, Nb^5+^, Mo^4+,5+,6+^, Ru^5+,6+,7+,8+^, Ag^2+,3+^, W^6+^, Re^7+^, Sb^5+^, and Bi^4+,5+^. Both methods were applied to evaluate oxidation states and in the case of disagreeing results, the bond valence method was preferred. Screening by the reducible specie oxidation state narrowed the candidates of interest from 29,829 to 16,582 materials.

### Insertion Site Finding

The next tier of screening candidate materials considers the identification of Mg insertion sites. Recently, Shen et al. reported a new approach for identifying the location of insertion sites in any given crystal structure, which will be referred to as the “insertion algorithm.”^38^ The insertion algorithm uses the calculated charge density of the material to identify charge density minima, which were shown to correlate strongly with viable insertion sites in known electrode materials. For each possible insertion site, the working ion of interest (such as Mg in this case) is inserted with one working ion per unit cell. A DFT relaxation is then performed to refine the site location and evaluate the change in the lattice and crystal structure to assess the possibility of viable insertion (whether the host structure is retained after insertion). As this tier requires multiple DFT calculations per material, it was not viable to apply the insertion algorithm to all 16,582 candidate materials at the time this work was performed. To demonstrate the screening workflow, the insertion algorithm was applied to a random selection of 2361 materials. Given the investigation of empty cathodes that are more stable in their charged state, the insertion algorithm was set to explore the possibility of a single working ion per unit cell as a first necessary requirement. Further successive working ion insertions can be repeated until there is significant host structural change or the minimum redox state of the compound is reached, thus determining the maximum intercalation level.

Of the 2361 materials where the insertion algorithm was applied, 1767 were discarded because the host structure changed significantly upon insertion. Additional properties useful for screening become computable after completing the insertion algorithm for a material. These properties include the voltage and relative stability (energy above hull) of the partially/fully magnesiated compound. The remaining 594 candidate materials were prioritized for the last tier of evaluating Mg^2+^ solid-state mobility using the following criteria: (1) average voltage >1.5 V, (2) energy per atom above the convex hull of the charged (unmagnesiated) material <0.05 eV/atom, and (3) energy per atom above the convex hull of the discharged (magnesiated) material <0.1 eV/atom. After applying these criteria, the number of candidates was further reduced by using the structure matching capabilities in pymatgen^44^ to select one material that would be representative of each unique structure type. As a result, 78 materials were selected for the next tier.

Applying the insertion algorithm to a host material produces a list of valid insertion sites for the working ion. These sites can be used to form a migration graph representing an interconnected network of Mg sites in the material, as introduced in our previous work.^46^ Sets of neighboring Mg sites can be extracted from this migration graph, which corresponds to a segment of a possible Mg^2+^ migration pathway in the material. Images representing the Mg^2+^ in various positions along these pathway segments can be generated and paired with DFT calculations in order to evaluate the energetics along the pathway segment. This information provides a key input for evaluating Mg^2+^ solid-state mobility in a given material, which is addressed in the following section.

### Multivalent Ion Solid-State Mobility

The last screening tier estimates the minimum energy barrier required for Mg^2+^ to migrate through the material and is the most computationally expensive tier. An upper limit migration barrier of 650 meV would remove materials, which exhibit sluggish intrinsic ionic mobility and provide a possibility for good rate capability that might allow for a C/2 cycling rate with nanosized particles.^36^ Given the evaluation of Mg-free compounds, only mobility in the charged (deintercalated) state at the dilute lattice limit was considered. Supercells were generated using pymatgen^44^ to avoid fictitious self-interaction effects from a neighboring Mg^2+^ due to periodic boundary conditions. Finally, the ApproxNEB algorithm^35^ implemented through the python package, atomate,^47^ was used to evaluate the migration barrier for a given pathway segment. The ApproxNEB algorithm was selected over the traditional NEB scheme due to its lower computational cost and robustness, which makes it more appropriate for high-throughput applications.^35^ Initial benchmarking work to compare the ApproxNEB algorithm to NEB for three known Mg cathode systems found that ApproxNEB required ∼5% of the computational resources compared to NEB (determined by considering the product of the number of nodes and wall time for running all calculations) and predicted energetic barriers within 150 meV of the NEB calculated values. Implemented in atomate, the ApproxNEB algorithm performs a series of relaxations for host, end point, and image structures for the specified migration events in a material. The key difference between NEB and ApproxNEB is in how the image relaxations are handled. With ApproxNEB, the images are relaxed independently of each other, and a coherent mobile ion path is maintained by fixing the positions of two atoms (the mobile working ion and the atom furthest away) in each image relaxation. Given the constraints of the ApproxNEB image relaxations, this method is likely to provide a slight overestimation of the energy barrier as compared to NEB. The energies produced by the ApproxNEB algorithm were mapped back onto the connections in the migration graph for a material. Pathway segments with incomplete points where calculations failed to reach sufficient convergence were excluded. The migration graph populated with the available energy information was used to locate the migration pathway through a material with the lowest barrier.

### ApproxNEB Calculation Details

The Vienna Ab initio Software Package was used to perform DFT calculations where the exchange correlation was approximated with the Perdew–Burke–Ernzerhof generalized gradient approximation (GGA). Pseudopotentials were selected according to “MPRelaxSet” specified in pymatgen.^44^ A U term was not included in these calculations as there is no conclusive evidence that GGA + U performs better when investigating ion migration with methods such as NEB.^20,48−50^ The total energy was sampled using a Monkhorst–Pack mesh with *k*-point density of 64 Å^–3^. Projector augmented-wave theory combined with a well-converged plane-wave cutoff of 520 eV were used to describe the wave functions. The convergence threshold of the total energy was set to 0.0005 eV and a force tolerance of 0.05 eV/Å.

## Results

**Figure 2:**
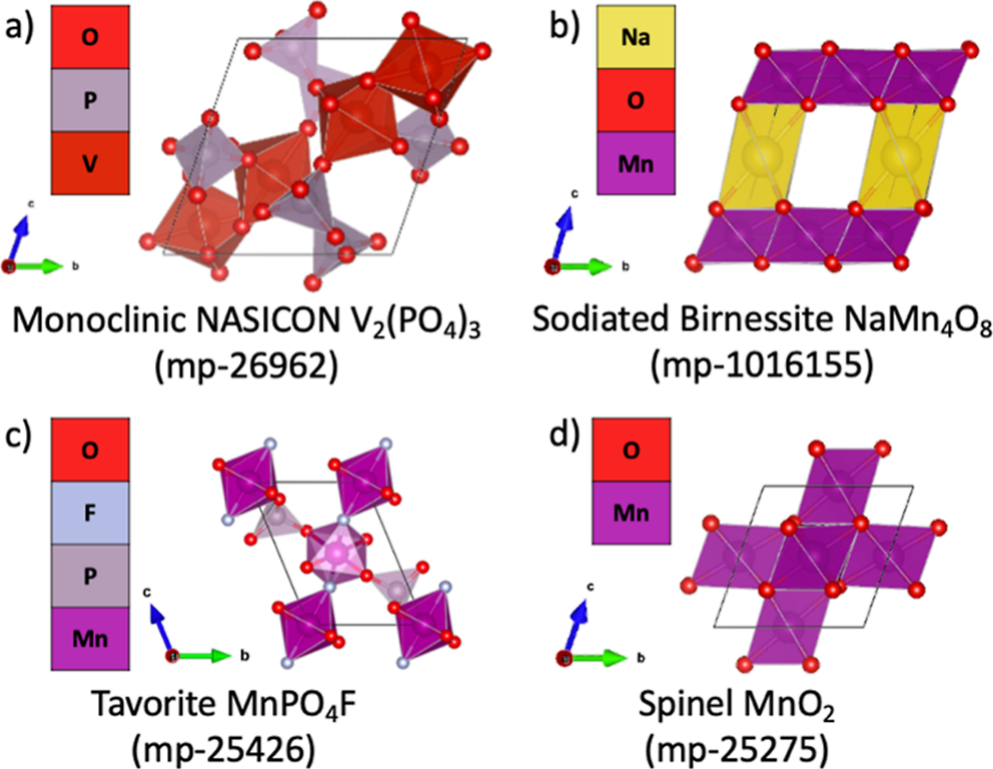
Unit cell crystal structures of each highlighted material in their unintercalated form are shown, including identifiers from the Materials Project: (a) monoclinic NASICON V_2_(PO_4_)_3_ from mp-26962, (b) sodiated birnessite NaMn4O8 from mp-1016155, (c) tavorite MnPO_4_F from mp-25426, and (d) spinel MnO_2_ from mp-25275.

**Figure 3:**
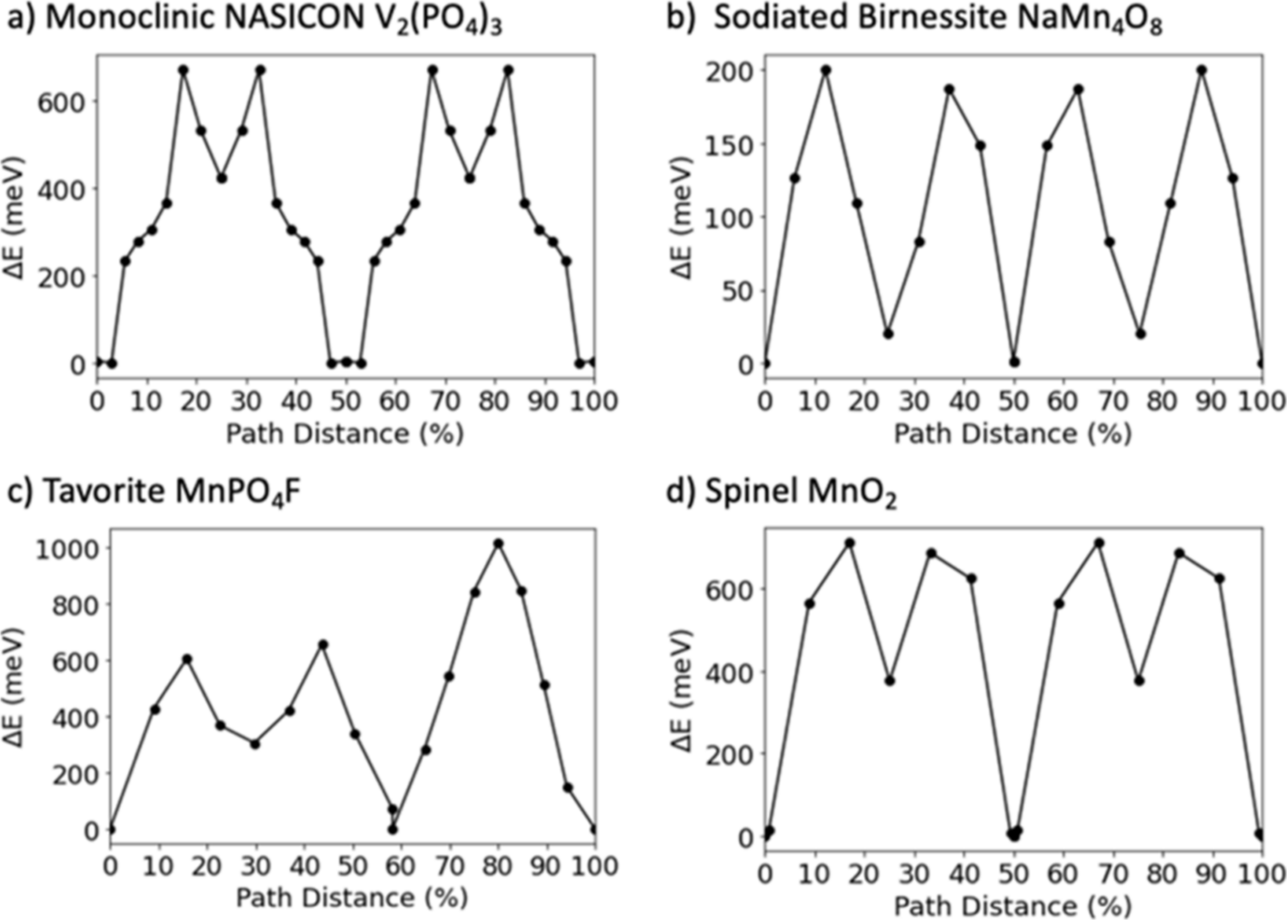
Plots of the energy landscape of Mg^2+^ migration along a percolating path for the four candidate materials. The percolating path starts from a given Mg site in a unit cell to the equivalent site in a neighboring unit cell, and the energy evolution along the path is predicted with ApproxNEB at the dilute lattice limit (single Mg in the host material supercell). Pathways were reduced to symmetrically distinct hops, which were calculated separately and then mapped back to the pathway for computational efficiency by avoiding redundant calculations. Lines connecting adjacent points are provided as a guide to the eye. (a) Monoclinic NASICON V_2_(PO_4_)_3_ (mp-26962) has an energetic barrier of 671 meV along a total path distance of 13.2 A. (b) Sodiated birnessite NaMn_4_O_8_ (mp-1016155) has an energetic barrier of 200 meV along a total path distance of 6.8 A. (c) Tavorite MnPO_4_F (mp-25426) has an energetic barrier of 1015 meV along a total path distance of 7.8 A. (d) Spinel MnO_2_ (mp-25275) has an energetic barrier of 711 meV along a total path distance of 7.4 A.

From the data set generated by the described screening process, 14 candidate materials were found to exhibit viable pathways for Mg^2+^ migration (ApproxNEB estimated barriers <800 meV). A few of these materials have been identified as novel and are currently being pursued electrochemically. In this section, we discuss in detail four materials as a representative set to showcase the diversity of crystal structures, energy content, site topology, and percolation pathways that can be assessed with this novel screening methodology. These four materials, V_2_(PO_4_)_3_ (mp-26962), NaMn_4_O_8_ (mp-1016155), MnPO_4_F (mp-25426), and MnO_2_ (mp-25275) which are depicted in Figure 2, are highlighted here as candidate Mg cathodes to illustrate the value of this screening approach. Table 1 summarizes the following properties of these cathodes: voltage, capacity (upon a single Mg insertion), charge (deintercalated) stability, discharge (intercalated) stability, and the estimated ApproxNEB energetic barrier for Mg^2+^ migration. Figure 3 shows the migration energy landscape determined with the ApproxNEB algorithm for Mg^2+^ along the best percolating path identified.

**Table 1:** Summary of Electrode Properties for the Four Mg Cathodes That Will be Described in More Detail^a^

material	voltage (V)	capacity (mA h/g)	charge (deintercalated) stability (meV/atom)	discharge (intercalated) stability (meV/atom)	energetic barrier (dilute lattice limit) (meV)
monoclinic NASICON, Mg_(*x* < 0.5)_V_2_(PO_4_)_3_, mp-26962	3.3	67	23	0	671
sodiated birnessite, Mg_(*x* < 1)_NaMn_4_O_8_, mp-1016155	2.2	136	19	82	200
tavorite, Mg_(*x* < 0.5)_MnPO_4_F, mp-25426	3.7	148	42	62	1015
spinel, Mg_(*x* < 0.5)_MnO_2_, mp-25275	2.9	144	51	45	711

a The voltage reported is a theoretical voltage (V) calculated from the energy difference of the intercalation reaction (Δ*G*_rxn_ = −*nFV*). The theoretical capacity (*Q*) was calculated based on the atomic mass of the intercalated material (*M*) with *Q* = *nF*/*M* based on the composition listed in the first column of Table 1. *n* represents the number of electrons transferred (for Mg, *n* = 2), and F is Faraday’s constant. Stability values (energy per atom above the convex hull) were calculated using the MP2020Compatibility scheme^51^ and Materials Project database phase diagrams using pymatgen.^44^ The lowest energetic barrier for Mg^2+^ migrating along a percolating pathway calculated with ApproxNEB at the dilute lattice limit (single Mg in host material supercell) is listed.

**V_2_(PO4)_3_ (mp-26962)** is a monoclinic NASICON that can be obtained experimentally from its lithiated version, Li_3_V_2_(PO_4_)_3_. This material has been studied as a Mg cathode where XAS spectra showed a change in the vanadium oxidation state upon Mg intercalation;^52^ however, definitive evidence of reversible Mg intercalation with repeated cycling is yet to be reported.^53^ The voltage predicted by the screening process (3.3 V) compares well with the experimentally measured value of ∼3.0 V versus Mg/Mg^2+^. Due to the single Mg insertion explored here, the calculated capacity corresponds to a lower limit magnesiation level of Mg_(*x* < 0.5)_V_2_(PO_4_)_3_ and hence is below the experimentally reported capacity of ∼197 mA h/g for Mg_(*x* < 1.5)_V_2_(PO_4_)_3_. Sufficient Mg^2+^ mobility for acceptable rate capability appears to be possible with an ApproxNEB predicted migration barrier of 671 meV. While this value is greater than the 650 meV threshold, as stated previously, ApproxNEB is known for overestimating the energy barrier; hence, it is likely that the true activation energy for dilute Mg^2+^ migration is lower. The results of this screening recommends further investigation of V_2_(PO_4_)_3_ as a Mg cathode, particularly highlighting the need for testing in electrolytes that are stable at sufficiently high voltages.

**NaMn_4_O_8_ (mp-1016155)** is a sodiated version of birnessite, δ-MnO_2_, which is the layered polymorph of MnO_2_. Birnessite is typically hydrated with water molecules between layers of MnO_6_ octahedra and has been studied in the literature as a Mg cathode.^53−55^ More success has been found using aqueous electrolytes where there is a reversible transformation from δ-MnO_2_ to λ-MnO_2_ upon discharge.^54^ The inclusion of crystalline water increases the interlayer spacing,^56^ which could be favorable for ion mobility; however, involving water presents a challenge for using birnessite in high-energy-density Mg batteries given the incompatibility of water with magnesium metal anodes. However, pillaring the structure with sodium ions instead of water molecules avoids these compatibility issues and may help explain the good Mg^2+^ mobility predicted by a low ApproxNEB migration barrier of 200 meV. Alkali-ion pillaring has been examined as a strategy to improve the electrochemical performance in vanadium oxides as Li-ion cathodes by stabilizing the structure and improving ion mobility.^57−59^ More detailed studies would be required to understand the role of sodium and possible avenues for facilitating multivalent ion mobility such as in NASICONs where sodium reordering upon calcium transport has been demonstrated.^12,13,60^ Replacing water with sodium in the birnessite structure could offer an avenue for using the layered δ-MnO_2_ polymorph as a cathode with magnesium metal anodes. While the synthesis of various sodiated versions of birnessite has been reported,^61^ electrochemical investigation has been limited to consideration as a Na cathode.^56,62,63^

**MnPO_4_F (mp-25426)** belongs to the tavorite structure family. This class of materials has been studied as Li-ion cathodes and includes examples such as LiVPO_4_F,^64^ LiFePO_4_F,^65^ and LiFeSO_4_F.^66^ Theoretical work on FeSO_4_F^67^ and VPO_4_F^68^ as Mg cathodes has been reported and found promising properties.^53^ However, no Mg^2+^ electrochemical experimental work has yet been published on these materials. With voltages of 2.5 V for FeSO_4_F and 2.6 V for VPO_4_F, perhaps difficulties in identifying higher voltage magnesium electrolytes present a roadblock. With a predicted voltage of 3.7 V for MnPO_4_F, the lack of suitable electrolytes is also a limitation that must be overcome before this material can be pursued experimentally. While reasonable Mg^2+^ migration barriers have been identified for other tavorites (360 meV for FeSO_4_F and 704 meV for VPO_4_F), the dilute lattice limit ApproxNEB barrier of 1015 meV for MnPO_4_F is prohibitively high. Although MnPO_4_F is an attractive cathode candidate from an energy density perspective, its poor solid-state Mg^2+^ mobility would result in poor rate capability.

**MnO_2_ (mp-25275)** is the spinel polymorph or the λ phase of MnO_2_. Given that spinel LiMn_2_O_4_ is a well-studied, commercialized lithium-ion cathode,^69^ there has been significant interest in studying spinels as Mg cathodes.^20,36,70^ While promising from an energy density standpoint with a voltage of 2.9 V versus Mg/Mg^2+^ and theoretical capacity of 144 mA h/g, it has been challenging to experimentally realize a high capacity with repeated cycling because of sluggish Mg^2+^ solid-state mobility.^53^ High Mg^2+^ migration barriers of ∼800 meV for the dilute lattice limit (charged/deintercalated state) calculated with NEB have been reported for spinel MgMn_2_O_4_,^20,36^ which is consistent with the high (>650 meV) migration barrier of 711 meV predicted by ApproxNEB in this screening method. This λ-MnO_2_ spinel example demonstrates the value of the fourth screening tier where ApproxNEB is used to estimate migration barriers to identify materials where Mg^2+^ solid-state mobility will be a challenge.

## Discussion

**Figure 4:**
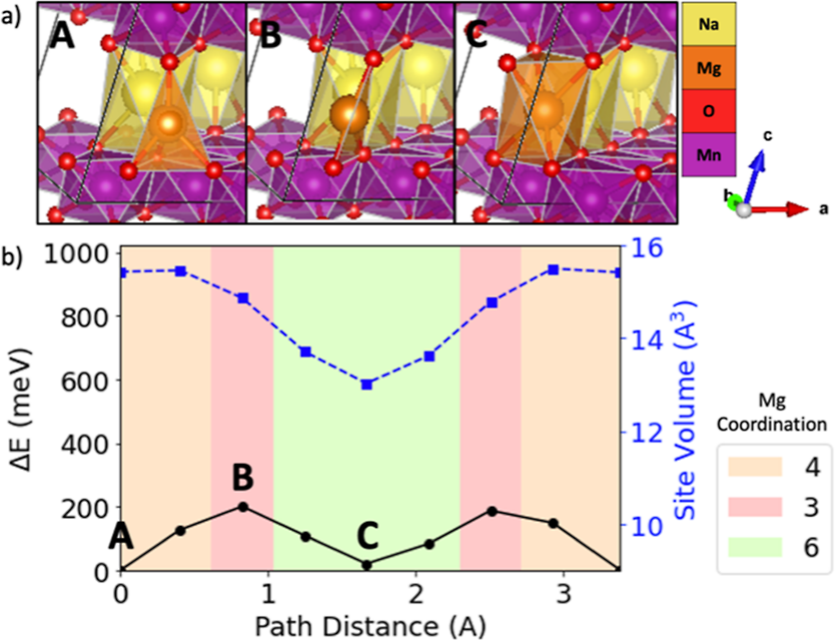
Evolving environment and associated Mg^2+^ migration energy as a function of the pathway coordinate in sodiated birnessite NaMn_4_O_8_ where (b) depicts a graph of the energy barrier (black line with circles), Mg site volume (blue line with squares), and Mg^2+^ coordination (colored graph area) based on ApproxNEB and (a) shows images of the Mg^2+^ at various positions along the migration pathway (labeled by A, B, and C).

**Figure 5:**
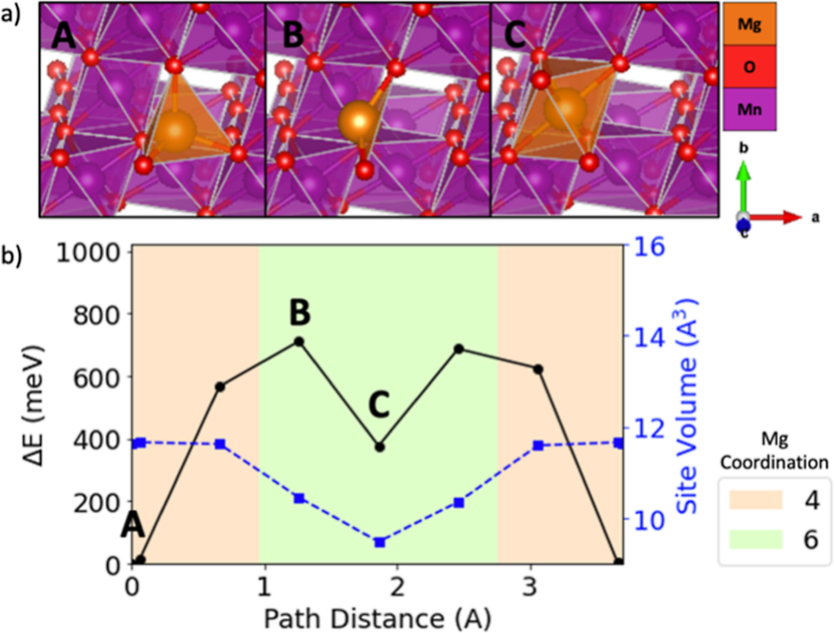
Evolving environment and associated Mg^2+^ migration energy as a function of the pathway coordinate in spinel MnO_2_ where (b) depicts a graph of the energy barrier (black line with circles), Mg site volume (blue line with squares), and Mg^2+^ coordination (colored graph area) based on ApproxNEB and (a) shows images of the Mg^2+^ at various positions along the migration pathway (labeled by A, B, and C).

**Figure 6:**
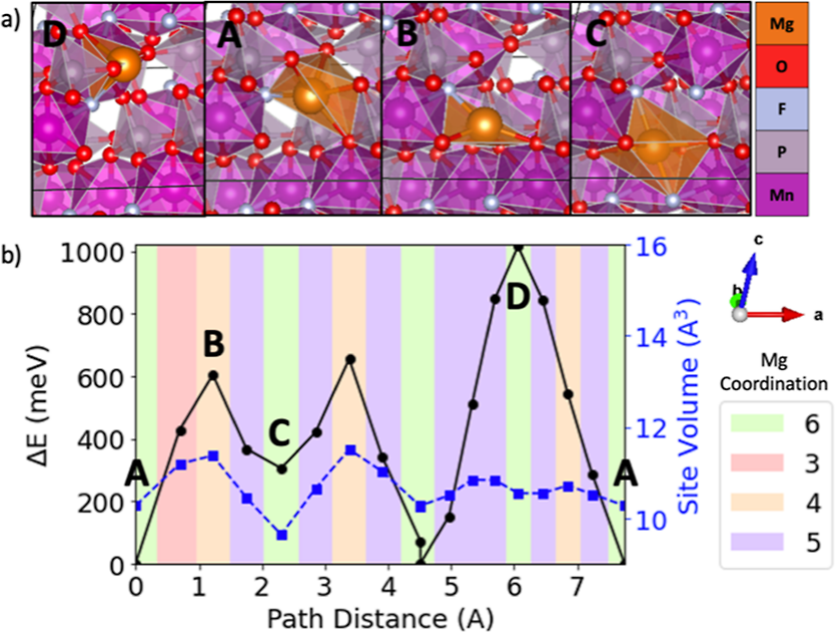
Evolving environment and associated Mg^2+^ migration energy as a function of the pathway coordinate in tavorite MnPO_4_F where (b) depicts a graph of the energy barrier (black line with circles), Mg site volume (blue line with squares), and Mg^2+^ coordination (colored graph area) based on ApproxNEB and (a) shows images of the Mg^2+^ at various positions along the migration pathway (labeled by A, B, C, and D).

**Figure 7:**
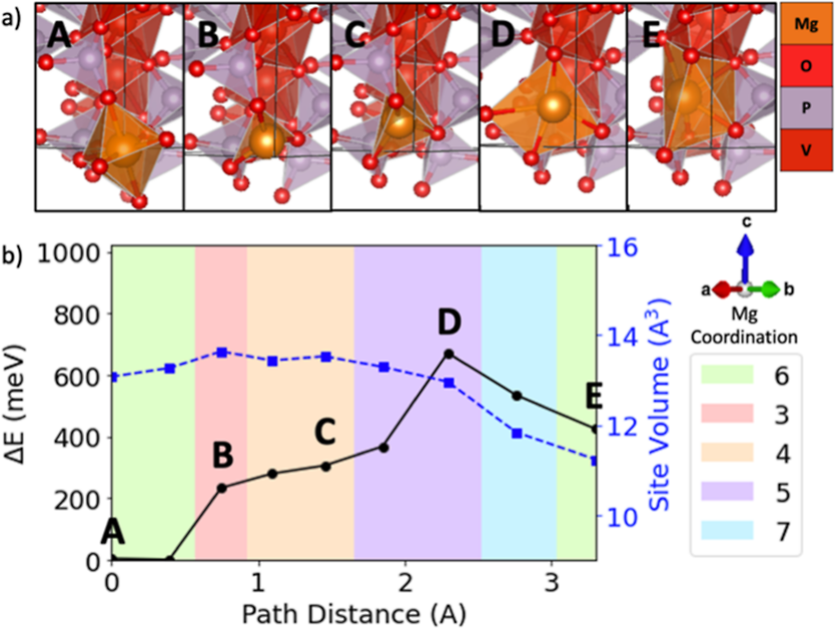
Evolving environment and associated Mg^2+^ migration energy as a function of the pathway coordinate in NASICON V_2_(PO4)_3_ where (b) depicts a graph of the energy barrier (black line with circles), Mg site volume (blue line with squares), and Mg^2+^ coordination (colored plot area) based on ApproxNEB and (a) shows the images of the Mg^2+^ at various positions along the migration pathway (labeled by A, B, C, D, and E).

The migration pathways and relaxed ApproxNEB image structures were examined in more detail for the four highlighted Mg cathode candidates: NASICON V_2_(PO_4_)_3_ (mp-26962), birnessite NaMn_4_O_8_ (mp-1016155), tavorite MnPO_4_F (mp-25426), and spinel MnO_2_ (mp-25275). In addition to mapping the energy difference along the pathway, the volume associated with the mobile Mg^2+^ calculated using the Voronoi algorithm through pymatgen is included.^44,71−73^ The graph area is colored to reflect the coordination of the Mg^2+^ at various positions along the path based on the relaxed ApproxNEB images, where the coordination number was analyzed using the CrystalNN algorithm in pymatgen.^44,71^ Images of the mobile Mg^2+^ in the host crystal structure made using VESTA software at significant points along the migration path are marked by letters and displayed with these plots in Figures 4–7.

A common feature in the pathways examined is the occurrence of high-energy (bottleneck) ion positions where the energy difference is the highest [e.g., Figure 4 shows position B for sodiated birnessite NaMn_4_O_8_, Figure 5 shows position B for spinel MnO_2_, Figure 6 shows position B and D for tavorite MnPO_4_F, and Figure 7 shows position B and D for NASICON V_2_(PO4)_3_] as the mobile Mg^2+^ passes through a plane of anions. In the case where the Mg^2+^ is moving from a tetrahedral to an octahedral site where the tetrahedra and octahedra are face sharing, this highest-energy point corresponds to the Mg^2+^ squeezing through the triangle of anions composing the shared face. This finding is in line with previous work on spinels where the area of the anion triangle was expanded by substituting larger and more polarizable anion atoms in order to lower the energetic penalty for migration.^74^ One counterintuitive finding, however, is that these bottleneck migration ion events do not correspond with the lowest volume sites along the migration path. In the identified pathways of these four materials, these lowest volume sites occur when Mg^2+^ is in a six-fold site of favored coordination, indicating a site with particularly favorable Mg-anion coordination and correspondingly tight bond lengths. This suggests that volume alone may not be a meaningful descriptor, and more significant conclusions can be made by comparing volume across sites with similar local bonding environments.

The identified pathways for sodiated birnessite NaMn_4_O_8_ and spinel MnO_2_ are both composed of face-sharing tetrahedra (tet) and octahedra (oct). While these two materials exhibit similar tet–oct–tet coordination changes, sodiated birnessite NaMn_4_O_8_ exhibits a much lower Mg^2+^ ApproxNEB migration barrier (200 meV) compared to spinel MnO_2_ (711 meV). One possible explanation is that the sodium in birnessite expands the interlayer spacing, reduces the electrostatic interaction between Mg^2+^ and the oxygen layers, and increases the available volume along the pathway compared to spinel MnO_2_. Therefore, the energy penalty for Mg^2+^ migration is lowered because the Coulombic interactions between the mobile ion and host structure are weaker.

Examining the high-energy positions in tavorite MnPO_4_F illustrates the influence of the anion composition when considering Mg^2+^ passing through planes of anions. As shown in Figure 6, the higher-energy bottleneck position D corresponds to the mobile Mg^2+^ passing through a plane of four oxygen atoms. The Mg^2+^ moves through a plane comprising two oxygens and one fluorine at the lower energy bottleneck position B. At D, the Mg^2+^ ion passes through the oxygen plane off center while at position B, the Mg^2+^ ion is in the center of the anion triangle. Therefore, it is likely that this position discrepancy contributes to the higher energy of point D, but it is also possible that substituting fluorine for one oxygen in the anion plane reduces the energetic penalty.

Of the six identified bottleneck positions where the mobile Mg^2+^ ion passes through an anion plane in these four materials, only one case, point B of Figure 7 for NASICON V_2_(PO4)_3_, does not correspond to a local energy maximum. We hypothesize that the relatively larger available volume for the mobile Mg^2+^ in NASICON V_2_(PO4)_3_ is responsible for the improved migration energetics, even when moving through anion planes. Both NASICON V_2_(PO4)_3_ and sodiated birnessite NaMn_4_O_8_ exhibit large volume Mg sites (>13 A^3^), as compared to spinel MnO_2_ and tavorite MnPO_4_F (<12 A^3^). The Mg sites exhibit larger volumes (>13 A^3^) and relatively low-energy differences (<350 meV) when considering the NASICON V_2_(PO4)_3_ oct–tet transition (Figure 7 A → B → C) and the sodiated birnessite NaMn_4_O_8_ tet–oct transition (Figure 4 A → B → C). Thus, the tet and oct volumes are large enough to avoid a substantial energy penalty when the Mg^2+^ squeezes through the shared anion face. This suggests that perhaps there is a minimum anion triangle area where a costly energy penalty for Mg^2+^ migration can be avoided.

## Conclusions

A new comprehensive materials screening framework designed to identify promising multivalent cathodes among materials that do not contain the active intercalating ion has been implemented and applied to Mg cathode discovery. The screening consists of four tiers, each focusing on a different set of properties relevant for multivalent cathodes. In the first tier, all materials in the Materials Project were filtered by relative stability and composition. Then, candidate materials were filtered to include at least one reducible cation with a high enough oxidation state to permit intercalation. In the third tier, possible multivalent ion insertion sites were identified, and candidates were prioritized by voltage, charge stability, and discharge stability. In the fourth and final screening tier, multivalent ion solid-state mobility is evaluated using the ApproxNEB algorithm. A major advancement in the development of this computational screening framework is the capability to evaluate the solid-state mobility of any inorganic crystalline material with automated high-throughput methods.

From the reported work on Mg cathodes, four materials NASICON V_2_(PO_4_)_3_ (mp-26962), birnessite NaMn_4_O_8_ (mp-1016155), tavorite MnPO_4_F (mp-25426), and spinel MnO_2_ (mp-25275) were identified as possible Mg cathodes and discussed in more detail. Experimental reports on NASICON V_2_(PO_4_)_3_ and spinel MnO_2_ validate the evaluation of these materials with the developed computational screening framework. NASICON V_2_(PO_4_)_3_ and birnessite NaMn_4_O_8_ were identified as promising Mg cathodes that warrant further experimental investigation. Among these examples, different Mg^2+^ migration barriers were observed despite having similar changes in coordination along the pathway. Local energy maxima were found to correlate with site topology (such as passing through an anion plane) rather than the lowest site volume along the path. However, when comparing only the anion plane sites, materials which exhibit a larger area between anions (such as NASICON V_2_(PO_4_)_3_ and birnessite NaMn_4_O_8_) were found to reduce the energy penalty for Mg^2+^ migration. Therefore, the available free volume for a given type of the local bonding environment of a mobile ion site in the host structure was proposed as another influential factor for improving solid-state mobility.
